# Use of Extracorporeal Therapies to Treat Severe Caffeine Poisoning

**DOI:** 10.1111/hdi.70002

**Published:** 2025-06-24

**Authors:** Saeko Kohara, Yoshito Kamijo, Michiko Takai, Ryoko Kyan, Eiju Hasegawa, Takuya Shimane

**Affiliations:** ^1^ Department of Clinical Toxicology Saitama Medical University Saitama Japan; ^2^ Department of Critical Care Medicine and Trauma National Hospital Organization Disaster Medical Center Tokyo Japan; ^3^ Department of Drug Dependence Research National Institute of Mental Health, National Center of Neurology and Psychiatry Tokyo Japan

**Keywords:** caffeinated tablets, caffeine, extracorporeal blood purification, hemodialysis

## Abstract

**Introduction:**

Reflecting on recent reports suggesting the efficacy of extracorporeal blood purification, including hemodialysis, for severe caffeine poisoning, we conducted a retrospective 5‐year follow‐up study on acute caffeine poisoning in Japan particularly focusing on extracorporeal blood purification.

**Methods:**

Patients transported to emergency facilities between April 2016 and March 2021 after consuming large or excessive amounts of over‐the‐counter drugs and energy drinks containing caffeine as a primary ingredient were included. We collected information on demographic characteristics, medical history, the caffeine‐containing products consumed, clinical and laboratory findings, treatments, and outcomes using a questionnaire. We compared the data of patients who were treated with and without extracorporeal blood purification (blood purification vs. nonblood purification group). We also compared our findings with those of a previous related study.

**Findings:**

Seventy‐six patients were included. Patients in the blood purification group (*n* = 22) were significantly older (*p* = 0.02), ingested higher estimated doses of caffeine (*p* < 0.01), demonstrated higher pulse rates (*p* = 0.01), respiratory rates (*p* = 0.03), and serum glucose levels (*p* = 0.05) on admission, resulting in longer hospital stays (*p* < 0.01) than those in the nonblood purification group (*n* = 54). However, their backgrounds, clinical features on arrival, and clinical signs and symptoms during the clinical course were similar between this and the previous study. Compared to the previous study, this study had a higher percentage of patients who underwent extracorporeal blood purification (16.8% vs. 28.9%; *p* = 0.07), including hemodialysis (10.9% vs. 22.4%; *p* = 0.06); nevertheless, no statistically significant differences were observed in the prognoses between the studies.

**Conclusions:**

Currently, the use of extracorporeal blood purification including hemodialysis for the treatment of caffeine poisoning is being promoted in Japan; however, the decision to administer it mostly depends on the patients' ingested doses or vital signs, and not on the serum caffeine levels. Further studies are warranted to confirm whether extracorporeal blood purification improves the prognoses of patients with severe caffeine poisoning.

## 1 Introduction

In recent years, several Japanese studies, including case reports suggesting the efficacy of extracorporeal blood purification for severe caffeine poisoning [1, 2, 3], a case series recommending hemodialysis as the first choice among extracorporeal blood purification options [4], and a clinical study recommending a blood concentration of ≥ 140 mg/L as the induction criteria for hemodialysis [5], have been published. Thus, in this study, we aimed to investigate if extracorporeal blood purification is being promoted as a treatment for severe caffeine poisoning in Japan and evaluate the conditions for its administration.

In this follow‐up study supported by the Japanese Society of Clinical & Analytical Toxicology, data from the subsequent 5 years were evaluated, with a particular focus on extracorporeal blood purification.

## 2 Materials and Methods

### 2.1 Case Definition

This study included patients transported to emergency facilities between April 2016 and March 2021 after consuming large or excessive amounts of over‐the‐counter drugs and energy drinks that contained caffeine as the primary ingredient. Patients who reported consuming these caffeinated products, were witnessed using such products, or were found to possess residual products at the scene were included in this study. Patients who consumed large or excessive amounts of prescription drug mixtures or over‐the‐counter drugs containing pharmacologically active ingredients such as acetaminophen, as well as caffeine, and patients who simultaneously overdosed using other drugs were excluded from the study.

### 2.2 Data Collection

The data collection methods, questionnaire content, and definition of terms are consistent with the previous study. At the beginning of April 2022, we sent an e‐mail to 32 emergency facilities with employees (i.e., physicians, pharmacists, and researchers) who were members of the Japanese Society of Clinical & Analytical Toxicology, to solicit their participation in this study. We asked participants working in emergency facilities to share data regarding patients. Participating emergency facilities were mailed a questionnaire that contained items on the following: patient age and sex; medical history; commercial names and amounts of the caffeinated products consumed; caffeine dose; how they obtained the caffeinated products; the reasons for the overdoses; vital signs and laboratory data on arrival; clinical signs and symptoms; physical complications during the clinical course; treatments including ventilator use, hemodialysis, continuous hemodiafiltration, continuous hemodialysis, hemoperfusion, and veno‐arterial extracorporeal membrane oxygenation; treatment outcomes; and results of blood toxicological analyses. The pilot questionnaire was assessed to ensure simplicity of understanding and the time required for completion. Physicians working in the participating emergency facilities were asked to identify all patient cases that satisfied the above conditions using information available inpatient medical records and databases, and to complete the questionnaire for every case they identified. All questionnaires were collected and analyzed at the Department of Clinical Toxicology, Saitama Medical University Hospital (the core facility of this study).

### 2.3 Definition of Terms

Tachypnea was defined as a respiratory rate ≥ 20 breaths/min; depressed consciousness as a Glasgow Coma Scale score of 3–14; tachycardia as a heart rate ≥ 100 beats/min; and hyperthermia as a body temperature ≥ 37.5°C. Hypercreatinekinasemia was defined as a serum creatine kinase concentration ≥ 200 IU/L; hyperglycemia as a serum glucose concentration ≥ 180 mg/dL; hypokalemia as a serum K+ concentration ≤ 3.4 mmol/L; hypophosphatemia as a serum inorganic phosphate concentration ≤ 2.4 mg/dL; and hyperlactatemia as a serum lactate concentration ≥ 2.0 mmol/L. Acute kidney injury was defined as a serum creatinine concentration ≥ 0.3 mg/dL or a 1.5‐fold increase in serum creatinine concentration following arrival at the facility. Liver injury was defined as an alanine aminotransferase concentration ≥ 80 IU/L (i.e., twice the normal upper limit). Acute respiratory distress syndrome was defined as follows: acute onset, bilateral pulmonary infiltrates consistent with the presence of edema, and impaired oxygenation with a PaO_2_/FiO_2_ ratio ≤ 200 in the absence of clinical evidence of left atrial hypertension. Rhabdomyolysis was defined as a creatine kinase concentration ≥ 10,000 IU/L (i.e., 50× the normal upper limit) with myoglobinuria.

### 2.4 Statistical Analyses

The demographic and clinical characteristics of the patients who overdosed on caffeinated products were analyzed. Based on whether or not they underwent extracorporeal blood purification, patients were divided into blood purification and nonblood purification groups, respectively. The Wilcoxon rank sum test and Fisher's exact test were used to compare the (a) epidemiological and clinical characteristics of patients in the two groups and (b) findings of this study with those of the previous study. The thresholds for statistical significance and trend were *p* < 0.05 and *p* < 0.10, respectively. Data were analyzed retrospectively using IBM SPSS Statistics, version 24 (IBM Corp., Armonk, NY, USA).

## 3 Results

### 3.1 Study Population

A total of 110 patients from 11 emergency facilities in Japan were evaluated. We excluded 31 patients who consumed large or massive amounts of over‐the‐counter drugs that contained pharmacologically active ingredients other than caffeine, one who simultaneously ingested large amounts of other drugs alongside caffeine (including benzodiazepines), and two who were transported to hospitals by ambulance outside of the study period. Ultimately, 76 patients were included in the study. The annual numbers of patients were 12 (April 2016 to December 2016), eight (January 2017 to December 2017), 10 (January 2018 to December 2018), 19 (January 2019 to December 2019), 21 (January 2020 to December 2020), and six (January 2021 to March 2021).

### 3.2 Patient Demographics

Most patients were younger (mean age, 25.3 ± 8.9 years; median age, 23 years; range, 15–54 years). Thirty‐seven patients (48.7%) were male and 39 (51.3%) were female. Among all the patients, 36 (47.4%) had a history of psychiatric consultation, with diagnoses of depression in 14 (18.4%), schizophrenia in four (5.3%), bipolar disorder in three (3.9%), personality disorder in three (3.9%), and other psychiatric diagnoses in 12 (15.8%). The reasons for the caffeine overdoses included suicide attempts or self‐injury in 65 patients (85.5%), other reasons in nine (11.8%), and no reason in two (2.6%). Seventy‐four patients (97.4%) consumed highly caffeinated tablets, one (1.3%) consumed highly caffeinated powder, and one (1.3%) consumed at least one highly caffeinated product (although it remained unclear whether this was in tablet or powder form). Among the 74 patients who consumed caffeinated tablets, five consumed more than one product, and nine consumed caffeinated liquids. Based on publicly available information regarding the commercial names and consumed amounts of caffeinated products, the mean caffeine dose ingested by 75 of the patients (98.7%) was estimated to be 9.6 ± 10.0 g (median, 7.0 g; range, 0.6–68.0 g). The caffeinated products were obtained online by 26 patients (34.2%), from stores such as drugstores and convenience stores by 22 (28.9%), from multiple sources by one (1.3%), by mail order by one (1.3%), and from unspecified sources by 26 (34.2%). Information regarding caffeine was obtained from the internet by 32 (42.1%) patients, and from unspecified sources by 44 (57.9%). The median interval between caffeine consumption and hospital arrival was estimated to be 3.3 h (range, 0.5–21 h) in 72 patients (94.7%). Serum caffeine concentrations on admission were determined for only two patients (2.6%; 240.9 and 178.0 μg/mL).

### 3.3 Clinical Manifestations

Many patients exhibited tachypnea (64.8%), tachycardia (51.3%), depressed consciousness (48.7%), hypokalemia (85.3%), hyperlactatemia (59.7%), hypophosphatemia (54.1%), hyperglycemia (37.7%), and hypercreatinekinasemia (36.5%) on arrival at the emergency department. The most common signs and symptoms (> 10%) during the patients' clinical courses were gastrointestinal symptoms such as nausea (65.8%) and vomiting (88.2%), consciousness disorders (31.6%), excitement or agitation (23.7%), tremor (14.5%), and arrhythmias (26.3%) including sinus tachycardia (23.7%) and ventricular tachycardia (3.9%), and 2 (2.6%) developed cardiac arrest. The only major organ complication that was noted was rhabdomyolysis (23.7%).

### 3.4 Treatments and Outcomes

Twenty‐two patients (28.9%) received extracorporeal blood purification, including hemodialysis (22.4%), hemoperfusion (2.6%), continuous hemodialysis, or continuous hemodiafiltration (5.3%). Among them, one patient received both hemoperfusion and continuous hemodiafiltration. Ten patients (13.2%) required ventilatory support. No patient underwent veno‐arterial extracorporeal membrane oxygenation. Thirty‐nine patients (51.3%) were treated with medication including metoclopramide (25%), potassium products (18.4%), benzodiazepines (11.8%), and others.

Sixty‐eight patients (89.5%) were hospitalized for a median of 3 days (range, 1–40 days). Among them, two patients (2.6%) who overdosed on highly caffeinated products in suicide attempts (caffeine doses of 36 and 14 g) developed cardiac arrest after arriving at the hospital. Seventy‐five patients (98.7%) recovered completely, while one (1.3%) was discharged with abdominal discomfort.

### 3.5 Comparison Between the Data of the Blood Purification Group (*n* = 22) and Those of the Nonblood Purification Group (*n* = 54)

The demographic characteristics and outcomes of 22 patients who were treated with extracorporeal blood purification (blood purification group) were compared with those of 54 patients who were not (nonblood purification group) (Table 1). Patients in the former group were significantly older than those in the latter group (*p* = 0.02). Estimated caffeine doses of patients in the former group were significantly higher (*p* < 0.01). Hospital stay of patients in the former group was significantly longer (*p* < 0.01).

**TABLE 1:** Demographic characteristics and outcomes of patients (*N* = 76).

	Blood purification group (*n* = 22)			Nonblood purification group (*n* = 54)			*p*
	*n* (%)	Median	IQR	*n* (%)	Median	IQR	
Age		26.0	22.8–32.3		21.5	18.0–27.3	0.02
Gender							0.31
Male	13 (59.1)			24 (44.4)			
Female	9 (40.9)			30 (55.6)			
Estimated caffeine dose (g)		12.0	8.0–20.0		5.0	2.4–10.0	< 0.01
Duration (min)		180.0	117.5–690.0		200.0	120.0–430.0	0.91
Length of hospitalization (day)		6.0	2.0–9.0		2.0	2.0–4.0	< 0.01
Patient outcomes							0.29
Residual symptoms	1 (4.5)			0			
Complete recovery	21 (95.5)			54 (100)			

*Note:* Duration = estimated time between ingestion of caffein‐containing products and arrival at emergency facility.

Abbreviation: IQR, interquatile range.

Vital signs and laboratory data on arrival at the hospital were compared between the two groups (Table 2). Laboratory data with a high frequency of abnormal values were extracted. Pulse rates (*p* = 0.01), respiratory rates (*p* = 0.03), and serum glucose levels (*p* = 0.05) were significantly higher in the former group. There were statistical trends for depressed GCS (*p* = 0.07) and elevated diastolic blood pressure (*p* = 0.07) in the former group.

**TABLE 2:** Comparison in vital signs and laboratory data on arrival at the hospital (*N* = 76).

	Blood purification group (*n* = 22)		Nonblood purification group (*n* = 54)		*p* ^a^
	Median	IQR	Median	IQR	
Vital signs					
Glasgow Coma Scale score	14.0	10.0–15.0	15.0	14.0–15.0	0.07
Systolic blood pressure (mmHg)	125.0	110.5–140.5	123.0	110.0–134.5	0.34
Diastolic blood pressure (mmHg)	77.5	73.0–84.0	73.0	65.0–82.0	0.07
Body temperature (°C)	36.5	36.0–37.1	36.5	36.2–36.8	0.99
Pulse rate (beats/min)	110.0	97.3–136.5	96.0	81.0–110.0	0.01
Respiration rate (breaths/min)	24.0	20.0–30.0	20.0	18.0–24.0	0.03
Laboratory data					
Creatine kinase (IU/L)	150.0	99.0–1176.3	120.0	89.0–293.8	0.30
Glucose (mg/dL)	188.5	142.5–218.0	158.0	129.0–182.0	0.05
K^+^ (mEq/L)	2.9	2.4–3.3	3.1	2.6–3.4	0.31
Lactic acid (mmol/dL)	3.45	0.71–6.43	2.78	0.51–5.55	0.39

Abbreviation: IQR, interquatile range.

^a^ Wilcoxon rank sum test.

### 3.6 Comparison Between the Results of the Previous (April 2011 to March 2016) and Present (April 2016 to March 2021) Studies

The data of the 101 patients included in the previous study were compared with those of the 76 included in this follow‐up study (Table 3). In both studies, most patients were young adults (median ages of 23 and 25 years in the previous and present study, respectively). No sex‐based differences were noted between the studies. The median caffeine doses were similar, at 7.2 and 7.0 g in the previous and present studies, respectively. Most patients in both studies had consumed caffeinated tablets (96.0% and 97.4% in the previous and present studies, respectively). The incidence of abnormal vital signs and laboratory data on arrival were similar except for hyperlactatemia, as were the clinical signs and symptoms during the patients' clinical courses. A comparison of the treatments and outcomes between the studies revealed that a higher number of patients (twice the percentage) in the present study underwent extracorporeal blood purification than in the previous study with a statistical trend (*p* = 0.07) and that more than twice the percentage of patients in the present study underwent hemodialysis than in the previous study with a statistical trend (*p* = 0.06). No deaths were recorded among the two patients (2.6%) who developed cardiac arrest in this study. However, among the seven (6.9%) who developed cardiac arrest in the previous study, three (3.0%) died. Nevertheless, no statistically significant differences or trends were noted between the studies in terms of the patients' prognoses. [Correction added on 17 October 2025, after first online publication: The study period has been updated in the Abstract, Introduction, Section 2.1 Case Definition, Section 3.1 Study Population and Section 3.6 Comparison Between the Results.]

**TABLE 3:** Comparison of previous (April 2011 to March 2016) and present (April 2016 to March 2021) studies.

	Previous study	Present study	*p*
	(*n* = 101)	(*n* = 76)	
Age (years), mean (SD)	26.2 (8.0)	25.3 (8.9)	0.44
Gender men/women	53 (52.5%) / 48 (47.5%)	37 (48.7%) / 39 (51.3%)	0.62
Estimated caffeine dose (g), mean (SD)	17.9 (83.4)	9.6 (10.0)	0.39
The time intervals between caffeine consumption and arrival at hospital (hours), mean (SD)	6.0 (5.5)	5.7 (5.4)	0.81
Vital signs			
Tachypnea (respiratory rate ≥ 20 breaths/min)	55/96 (57.3%)	46/71 (64.8%)	0.33
Tachycardia (heart rate ≥ 100 beats/min)	58/98 (59.2%)	39/76 (51.3%)	0.30
Depressed consciousness (Glasgow Coma Scale score = 3–14)	36/98 (36.7%)	37/76 (48.7%)	0.11
Abnormal laboratory data			
Hypokalemia (K^+^ ≤ 3.4 mmol/L)	82/97 (84.5%)	64/75 (85.3%)	0.89
Hyperlactatemia (lactate ≥ 2.0 mmol/L)	25/74 (33.8%)	37/62 (59.7%)	< 0.01
Hypophosphatemia (inorganic phosphate ≤ 2.4 mg/dL)	27/46 (58.7%)	20/37 (54.1%)	0.67
Hyperglycemia (glucose ≥ 180 mg/dL)	43/91 (47.3%)	26/69 (37.7%)	0.23
Hypercreatinekinasemia (creatine kinase ≥ 200 IU/L)	39/94 (41.5%)	27/74 (36.5%)	0.51
Ventilator support	15 (14.9%)	10 (13.2%)	0.87
Extracorporeal blood purification	17 (16.8%)	22 (28.9%)	0.07
Hemodialysis	11 (10.9%)	17 (22.4%)	0.06
Hemoperfusion	4 (4.0%)	2 (2.6%)	0.70
CHD/CHDF	3 (3.0%)	4 (5.3%)	0.47
VA‐ECMO	2 (2%)	0 (0%)	0.51
Hospitalization	85 (84.2%)	68 (89.5%)	0.30
Length of hospitalization (days), mean (SD)	4.4 (5.2)	4.5 (5.7)	0.87
Cardiac arrest	7 (6.9%)	2 (2.6%)	0.30
Death	3 (3.0%)	0 (0%)	0.26
Complete recovery	97 (96%)	75 (98.7%)	0.39

Abbreviations: CHD, continuous hemodialysis; CHDF, continuous hemodiafiltration; VA‐ECMO, veno‐arterial extracorporeal membrane oxygenation.

## 4 Discussion

Caffeine (1,3,7‐trimethylxanthine) is a natural alkaloid that is derived from tea leaves, coffee beans, cocoa beans, and kola nuts [6]. Caffeine is present not only in green tea, black tea, and coffee, but also in energy drinks and over‐the‐counter drugs. After oral ingestion, caffeine is rapidly and almost completely absorbed from the gastrointestinal tract and mainly metabolized by the cytochrome P450 enzyme system (CYP1A2, 2E1, and 3A) [7] with a serum elimination half‐life of 2–10 h [8], which may be extended to approximately 15 h after large or excessive consumption owing to the saturated activity of metabolic enzymes [9, 10]. Hemodialysis as well as hemoperfusion may be theoretically effective in removing caffeine because it has a small molecular weight (194.2 g/mol), a small distribution volume (0.6 L/kg), and a relatively low protein binding rate (36%). In Japan, more than 300,000 patients receive maintenance hemodialysis because of chronic renal failure, which is equivalent to more than 2000 per million [11]. Therefore, hemodialysis is readily available at many medical facilities, and the cost is approximately 30,000 yen (200 dollars) per session, which is inexpensive compared with other modalities of treatment used in intensive care.

In Japan, highly caffeinated tablets that have been commercialized for weight loss or the prevention of drowsiness and fatigue have been introduced as “a useful method to die by suicide” on suicide websites and social networking sites, resulting in an increased number of patients with acute caffeine poisoning [12]. Kamijo et al. conducted a multicenter retrospective study on the epidemiological and clinical features of patients in the emergency department who had consumed massive amounts of caffeinated products in Japan between April 2011 and March 2016 [13]. Consequently, 101 patients who consumed 1.2–82.6 g of caffeine (median, 7.2 g) were included. Among them, 11 (10.9%) patients underwent hemodialysis, four (4.0%) hemoperfusion, and three (3.0%) continuous hemodialysis or continuous hemodiafiltration to facilitate caffeine excretion. Although 97 (96.6%) patients recovered completely, three patients (3.0%) died among seven patients (6.9%) who developed cardiac arrest after consuming ≥ 6.0 g of caffeine.

In this follow‐up study, 76 patients who consumed 0.6–68.0 g of caffeine (median, 7.0 g) were included. Among them, 17 (22.4%) patients underwent hemodialysis, two (2.6%) hemoperfusion, and four (5.3%) continuous hemodialysis or continuous hemodiafiltration. All patients recovered without serious sequelae or death, including two (2.6%) who developed cardiac arrest after arriving at the hospital and were successfully treated with extracorporeal blood purification. A comparison of the data between the blood purification and nonblood purification groups revealed that patients with significantly higher estimated doses of caffeine, and higher pulse rates, respiratory rates, and serum glucose levels underwent extracorporeal blood purification, resulting in significantly longer hospital stays. A comparison of the results of this study with those of the previous study revealed no significant differences in patient backgrounds (e.g., age, sex, and caffeine doses), clinical features (including vital signs and laboratory data on arrival) except for hyperlactatemia, and clinical signs and symptoms during patients' clinical courses. However, while there was a higher percentage of patients who underwent extracorporeal blood purification (16.8% vs. 28.9%; *p* = 0.07), including hemodialysis (10.9% vs. 22.4%; *p* = 0.06) in the present study, no significant differences were observed in the outcomes.

In recent years, more case reports concerning patients with severe caffeine poisoning who were successfully treated using extracorporeal blood purification including hemodialysis [1, 2, 3], clinical studies on the indications for hemodialysis [4, 5], and rapid analytical methods for caffeine [14, 15] have been published. Ishigaki et al. reported a case of a 32‐year‐old male patient who developed sustained ventricular tachycardia after ingesting approximately 15.6 g of caffeine. The patient was treated with a combined therapy of hemoperfusion and hemodialysis, resulting in rapid improvement of the arrhythmia as well as a decrease in plasma caffeine levels [1]. Yasuda et al. reported the case of a 43‐year‐old male patient who developed ventricular fibrillation after ingesting 20 g of caffeine. The patient was treated by veno‐arterial extracorporeal membrane oxygenation and hemodialysis, resulting in rapid improvement in clinical findings [2]. Mitsui et al. have reported the case of a 22‐year‐old male patient who developed refractory ventricular fibrillation after ingesting highly caffeinated tablets (40 g caffeine in total). Hemodialysis was performed for 11 h under circulatory support using veno‐arterial extracorporeal membrane oxygenation, which rapidly decreased his serum caffeine concentration from 454.9 μg/mL on admission to 55.5 μg/mL, resulting in significant improvements to his clinical symptoms and circulatory parameters [3].

Yoshizawa et al. have reported a case series of two 24‐year‐old male patients who exhibited severe signs and symptoms after ingesting highly caffeinated tablets. One was treated using hemodialysis with a blood flow rate of 200 mL/min and a dialysate flow rate of 500 mL/min. The other was treated using hemoperfusion with a blood flow rate of 150 mL/min. The authors compared the clearances of hemodialysis (79.3–102.3 mL/min) and hemoperfusion (67.4–75.5 mL/min) by determining the patients' serum caffeine concentrations before and after cartridges or dialyzers (filters), respectively. This case series suggests that, although the two treatments resulted in similar clearances, hemodialysis may be superior to hemoperfusion because hemodialysis has a lower incidence of complications such as thrombocytopenia, is notably less expensive, and has significantly more skilled technicians [4]. Yoshizawa et al. have also found, in their clinical study supported by the Japanese Society of Clinical & Analytical Toxicology, that the serum caffeine concentration that most clearly separated the group with severe caffeine poisoning (defined as a blood pressure ≤ 90 mmHg; heart rate ≥ 140 beats/min; Glasgow Coma Scale score ≤ 8; ventricular arrhythmia such as ventricular tachycardia; or convulsive seizures) from the group with nonsevere caffeine poisoning was 147.43 mg/L. [5] They recommend initiating hemodialysis to treat severe caffeine poisoning when patients exhibit a serum caffeine concentration ≥ 140 mg/L.

However, only a few emergency facilities in Japan can measure serum caffeine concentration within a short timeframe after the patient arrives. In the present study, serum caffeine concentrations on admission were not determined in most patients. The indications for extracorporeal blood purification, including hemodialysis, may have been determined not based on serum concentrations but on estimated doses and vital signs such as pulse rates. Further development of methods for rapidly measuring serum caffeine concentration may therefore be warranted. Hanazawa et al. and Usui et al. have separately developed methods for rapidly and accurately measuring serum caffeine concentration using liquid chromatography–tandem mass spectrometry, which may contribute to better real‐time evaluation of the severity of caffeine poisoning and the decision to introduce extracorporeal blood purification [14, 15].

Besides the theoretical effectiveness supported by pharmacokinetics, easy availability, and low cost in Japan, the case reports and studies originating from Japan may have promoted hemodialysis as a treatment for this condition in the country, as was suggested in the present study. The Japanese Society of Clinical & Analytical Toxicology has recommended the use of extracorporeal blood purification, including hemodialysis, for treating caffeine poisoning (Table 4). This recommendation may further promote this modality of treatment. In the present study, there was a trend of a higher number of patients receiving extracorporeal blood purification, including hemodialysis, but there was no significant difference or trend for improved outcomes compared with the previous study. To evaluate the effectiveness of extracorporeal blood purification, outcomes should be compared with or without hemodialysis, adjusting for background factors, preferably in a prospective study.

**TABLE 4:** Guidelines for extracorporeal blood purification use to treat caffeine poisoning recommended by Japanese Society of Clinical and Analytical Toxicology.

Extracorporeal blood purification should be considered to treat a patient with serum caffeine concentration ≥ 140 mg/L, cardiopulmonary arrest, circulatory collapse, severe tachycardia, consciousness disturbance, ventricular arrhythmia, or convulsive seizures.
2 Hemodialysis should be considered as first choice of extracorporeal blood purification.
3 Hemodialysis under circulatory support by veno‐arterial extracorporeal membrane oxygenation should be considered to treat a patient with lethal ventricular arrhythmia or cardiac arrest.
4 Continuous extracorporeal blood purification, including high flow continuous hemodialysis, should be considered to treat a patient too hemodynamically unstable to introduce hemodialysis.

## 5 Limitations

This study has certain limitations that are worth noting. There may have been some selection bias owing to the small number of emergency facilities that were included. Although two separate studies were compared and discussed in this report, it is important to note that they involved different participating facilities.

## 6 Conclusion

Recently, the use of extracorporeal blood purification, including hemodialysis, has been promoted for treating patients who ingest higher doses of caffeine, resulting in more abnormal vital signs. However, there is currently no evidence that it is effective for severe caffeine poisoning. Further studies are warranted to confirm whether it improves the prognosis of patients with severe caffeine poisoning.

## Ethics Statement

The study was conducted in accordance with the principles of the Declaration of Helsinki, and the protocol was approved by the institutional review board of the Ethics Committee of Saitama Medical University Hospital (approval no.: 2021‐064). The possibility of secondary data use was included in the ethical review of the previous study, and the secondary data use was approved in the protocol for the present study.

## Conflicts of Interest

The authors declare no conflicts of interest.

## Data Availability

The data that support the findings of this study are available from the corresponding author upon reasonable request.

## References

[1] S. Ishigaki , H. Fukasawa , N. Kinoshita‐Katahashi , H. Yasuda , H. Kumagai , and R. Furuya , “Caffeine Intoxication Successfully Treated by Direct Hemoperfusion and Hemodialysis,” Internal Medicine 53 (2014): 2745–2747. 25447662 10.2169/internalmedicine.53.2882

[2] S. Yasuda , M. Hisamatsu , T. Hirano , et al., “Caffeine Poisoning Successfully Treated by Venoarterial Extracorporeal Membrane Oxygenation and Emergency Hemodialysis,” Acute Medicine & Surgery 8, no. 1 (2021): e627. 33532077 10.1002/ams2.627 PMC7831216

[3] D. Mitsui , Y. Kamijo , T. Yoshino , T. Hanazawa , T. Yoshizawa , and F. Iwase , “Severe Caffeine Poisoning Treated With Intermittent Hemodialysis Under Circulatory Support,” American Journal of Emergency Medicine 76 (2024): 270.e5–270.e7. 10.1016/j.ajem.2023.12.014 38129271

[4] T. Yoshizawa , Y. Kamijo , T. Hanazawa , et al., “Which of Hemodialysis and Direct Hemoperfusion Is More Recommended for Treating Severe Caffeine Poisoning?,” American Journal of Emergency Medicine 37, no. 9 (2019): 1801–1802. 30876774 10.1016/j.ajem.2019.03.010

[5] T. Yoshizawa , Y. Kamijo , T. Hanazawa , and K. Usui , “Criterion for Initiating Hemodialysis Based on Serum Caffeine Concentration in Treating Severe Caffeine Poisoning,” American Journal of Emergency Medicine 46 (2021): 70–73. 33735699 10.1016/j.ajem.2021.03.017

[6] P. J. Rogers , N. J. Richardson , and N. A. Elliman , “Overnight Caffeine Abstinence and Negative Reinforcement of Preference for Caffeine Containing Drinks,” Psychopharmacology 120, no. 4 (1995): 457–462. 8539327 10.1007/BF02245818

[7] W. Tassaneeyakul , D. J. Birkett , M. E. McManus , et al., “Caffeine Metabolism by Human Hepatic Cytochromes P450: Contributions of 1A2, 2E1 and 3A Isoforms,” Biochemical Pharmacology 47 (1994): 1767–1776. 8204093 10.1016/0006-2952(94)90304-2

[8] G. B. Kaplan , D. J. Greenblatt , B. L. Ehrenberg , et al., “Dose‐Dependent Pharmacokinetics and Psychomotor Effects of Caffeine in Humans,” Journal of Clinical Pharmacology 37 (1997): 693–703. 9378841 10.1002/j.1552-4604.1997.tb04356.x

[9] C. Willson , “The Clinical Toxicology of Caffeine: A Review and Case Study,” Toxicology Reports 5 (2018): 1140–1152. 30505695 10.1016/j.toxrep.2018.11.002 PMC6247400

[10] C. L. Leson , M. A. McGuigan , and S. M. Bryson , “Caffeine Overdose in an Adolescent Male,” Journal of Toxicology. Clinical Toxicology 26 (1988): 407–415. 3193494 10.1080/15563658809167105

[11] I. Masakane , S. Nakai , S. Ogata , et al., “An Overview of Regular Dialysis Treatment in Japan (As of 31 December 2013),” Therapeutic Apheresis and Dialysis 19, no. 6 (2015): 540–574. 26768810 10.1111/1744-9987.12378

[12] M. Hirose , A. Hirakawa , W. Niwa , et al., “Acute Drug Poisoning Among Adolescents Using Over‐The‐Counter Drugs: Current Status,” Yakugaku Zasshi 141, no. 12 (2021): 1389–1392. 34853209 10.1248/yakushi.21-00147

[13] Y. Kamijo , M. Takai , Y. Fujita , and K. Usui , “A Retrospective Study on the Epidemiological and Clinical Features of Emergency Patients With Large or Massive Consumption of Caffeinated Supplements or Energy Drinks in Japan,” Internal Medicine 57, no. 15 (2018): 2141–2146. 29526946 10.2169/internalmedicine.0333-17 PMC6120846

[14] T. Hanazawa , Y. Kamijo , T. Yoshizawa , and K. Usui , “Rapid Measurement of Serum Caffeine Concentrations in Acute Clinical Settings,” Toxicology Communications 5, no. 1 (2021): 97–101.

[15] K. Usui , Y. Fujita , Y. Kamijo , Y. Igari , and M. Funayama , “LC‐MS/MS Method for Rapid and Accurate Detection of Caffeine in a Suspected Overdose Case,” Journal of Pharmacological and Toxicological Methods 107 (2021): 10694. 10.1016/j.vascn.2020.106946 33276087

